# Colitides

**DOI:** 10.1155/2011/607434

**Published:** 2011-12-18

**Authors:** Genevieve B. Melton, Mary R. Kwaan, Hejin Hahn, Christopher J. Shepela, Jean S. Wang, Elizabeth C. Wick

**Affiliations:** ^1^Department of Surgery and Institute for Health Informatics, University of Minnesota, Minneapolis, MN 55455, USA; ^2^Department of Surgery, University of Minnesota, Minneapolis, MN 55455, USA; ^3^Department of Pathology, Virginia Mason Medical Center, Seattle, WA 98101, USA; ^4^Department of Gastroenterology, Regions Hospital-The Digestive Center, University of Minnesota, Saint Paul, MN 55130, USA; ^5^Division of Gastroenterology, Washington University School of Medicine, St. Louis, MO 63110, USA; ^6^Department of Surgery, Johns Hopkins Medical Institutions, Baltimore, MD 21287, USA

Colitides remain an important yet less studied group of inflammatory colon and rectal conditions with a range of etiologies. This special issue includes a total of eight papers examining several of these entities and related clinical issues. Together, these papers highlight the breadth of these disorders and their treatment, as well as some of the cutting-edge issues related to our understanding of colitides and the relationship of colorectal inflammation and disease.

Perhaps the most prominent iatrogenic colitide is *Clostridium difficile* colitis, an increasingly prevalent and virulent condition. D. T. Rubin and colleagues conducted a prospective open-label pilot study with rifaximin for treatment of *Clostridium difficile *colitis, “*Rifaximin is effective for the treatment of clostridium difficile-associated diarrhea: results of an open-label pilot study*”. While Rifaximin demonstrated efficacy, this study highlights the need for further prospective work to further assess its role in *Clostridium difficile *colitis management. N. E. Burkart et al. examined the use of flexible lower endoscopy as a diagnostic modality to further define its utility and role in clinical care in conjunction with clinical examination and diagnostic stool studies in “*Indications and relative utility of lower endoscopy in the management of clostridium difficile infection*”. In contrast, P. Sinh and colleagues conducted a review of the literature looking at the association and outcomes of *Clostridium difficile* colitis and inflammatory bowel disease in “*Clostridium difficile infection and inflammatory bowel disease: a review*”.

 Three of these studies examine fundamental biomedical and diagnostic issues to colorectal inflammation. S. S. Yoon and J. Sun review our current understanding of probiotics and their role in anti-inflammatory pathways as shown in “*Probiotics, nuclear receptor signaling, and anti-inflammatory pathways*”. As our understanding colitides and inflammatory pathways increase, these mechanisms become more important. To better understand the link between colorectal inflammation and malignancy, Å. Håkansson and colleagues developed a rat model to better delineate mechanisms between chronic inflammation and transformation to malignancy in a model for colorectal oncogenesis as shown in “*Colorectal oncogenesis and inflammation in a rat model based on chronic inflammation due to cycling DSS treatments*”. Finally, K. Song et al. compared the use of a jumbo biopsy forceps in colonic surveillance, demonstrating objective evidence of improved sample size and potentially better biopsy specimen quality for lower endoscopy “*Novel jumbo biopsy forceps for surveillance of inflammatory bowel disease: a comparative retrospective assessment*”.

Two final papers from W. B. Gaertner et al. “*Eosinophilic colitis: university of minnesota experience and literature review*” and N. Do et al. “*Radiation proctitis: current strategies in management*”, are detailed reviews on two less common colitides. First, W. B. Gaertner performed a descriptive case series and a review of eosinophilic colitis. N. Do and colleagues examined radiation proctitis, looking at the presentation, signs and symptoms, and range of treatments for this entity. With the increasing shift towards radiation therapy for prostate cancer and gynecological malignancies, the incidence of radiation proctitis will likely continue to increase in our elderly populations.

We hope that you will find the content in this special issue to be a valuable resource. Ultimately, we look forward to bringing further attention to colitides within the greater gastrointestinal, colorectal, and pathology communities and to help inspire more investigators to further our understanding of these important conditions.

**Genevieve B. Melton** **Genevieve B. Melton** **Mary R. Kwaan** **Mary R. Kwaan** **Hejin Hahn** **Hejin Hahn** **Christopher J. Shepela** **Christopher J. Shepela** **Jean S. Wang** **Jean S. Wang** **Elizabeth C. Wick** **Elizabeth C. Wick**

